# New Insights into Synergistic Boosts in SCFA Production Across Health Conditions Induced by a Fiber Mixture

**DOI:** 10.3390/nu17243904

**Published:** 2025-12-13

**Authors:** Gabriel S. Galeano-Garcia, Tingting Chen, Phillip A. Engen, Ali Keshavarzian, Bruce R. Hamaker, Thaisa M. Cantu-Jungles

**Affiliations:** 1 Department of Food Science, Purdue University, West Lafayette, IN 47907, USA; 2 Whistler Center for Carbohydrate Research, Purdue University, West Lafayette, IN 47907, USA; 3 State Key Laboratory of Food Science & Technology, School of Food Science & Technology, Nanchang University, Nanchang 330047, China; 4 Rush Center for Integrated Microbiome and Chronobiology Research, Rush University Medical Center, Chicago, IL 60612, USA; 5 Department of Internal Medicine, Rush University Medical Center, Chicago, IL 60612, USA; 6 Department of Anatomy and Cell Biology, Rush University Medical Center, Chicago, IL 60612, USA; 7 Department of Physiology, Rush University Medical Center, Chicago, IL 60612, USA

**Keywords:** fiber mixtures, short-chain fatty acids, synergism, health and disease

## Abstract

**Background/Objectives:** Short-chain fatty acids (SCFAs) produced by gut microbiota from dietary fiber fermentation play crucial roles in health and disease. While most research focuses on individual fibers, this study investigated whether a fiber mixture could synergistically increase SCFAs, exceeding the expected average production of individual contributions and microbial signatures associated with this phenomenon. **Methods:** We quantified synergistic SCFA production using secondary analysis of in vitro fecal fermentation data from 33 participants across four health conditions (healthy controls, Crohn’s disease, ulcerative colitis, and Parkinson’s disease), by comparing observed fiber mixture output to expected additive contributions from individual components and identified microbial signatures associated with synergistic SCFA production through differential abundance and network analyses. **Results:** The fiber mixture consistently outperformed most individual fibers and demonstrated synergistic SCFA production, yielding 32.8 ± 20.1% more total SCFAs than expected. This synergistic effect was observed across all four health conditions studied, with many individuals showing >50% synergy and some exceeding 80%. Differential abundance analysis revealed that individuals exhibiting synergistic responses were enriched in taxa from *Lachnospiraceae* and *Ruminococcaceae* families, key butyrate-producing groups that likely facilitate cooperative interactions. Network analysis confirmed these families as central hubs in synergy-associated microbial interactions. **Conclusions:** These findings suggest that fiber mixtures can harness cooperative microbial interactions to synergistically enhance SCFA production regardless of health status, offering potential for developing more effective prebiotics for superior therapeutic outcomes.

## 1. Introduction

Short-chain fatty acids (SCFAs) are bacterial fermentation products that emerge from the microbial degradation of dietary fibers, carbohydrate polymers, and oligomers not hydrolyzed by endogenous enzymes in the small intestine [1]. These metabolites, mainly acetate, propionate, and butyrate, serve as critical signaling molecules that regulate inflammation, immune responses, and colonocyte metabolism [2,3], while providing protective effects against metabolic disorders and inflammatory diseases [4]. Reduced microbial metabolic capacity, particularly in disease conditions, often manifests in depleted SCFA levels and dysbiotic microbiota composition, contributing to barrier dysfunction and systemic inflammation across multiple physiological systems, making SCFA restoration a therapeutic priority [5].

Targeted dietary fiber interventions have emerged as a principal strategy to enhance microbial fermentation and thereby increase SCFA production [6]. However, despite growing evidence that dietary fermentation dynamics can be altered when fibers are administered as a mixture [7,8], current research has predominantly focused on evaluating single-fiber effects, while the collective effects of fiber mixtures on SCFA production remain unexplored [9]. This research bias toward single-fiber formulations overlooks the potential for emergent properties that may arise when structurally diverse dietary fibers are used in combination.

We recently demonstrated in healthy individuals that strategically designed fiber mixtures can exhibit synergistic effects, enhancing SCFA generation beyond the theoretical additive effects of individual components, while supporting superior alpha-diversity and reducing variability in responses across individuals [7]. These findings indicate that fiber mixture responses are not merely the average of individual components but rather reflect complex cooperative microbial interactions that remain poorly understood and it is unclear if such synergistic interactions would also occur across disease states.

The importance of fiber mixture interactions and their potential to promote enhanced SCFA production becomes particularly apparent when considering disease-associated dysbiosis. In this regard, Chen et al. [10] evaluated gut microbial communities in localized gut inflammation-related diseases including Crohn’s disease (CD) and ulcerative colitis (UC), as well as systemic Parkinson’s disease (PD), compared to healthy controls (HC), employing a four-component soluble fiber mixture of apple pectin, barley β-glucan, fructooligosaccharides, and sorghum arabinoxylan [10]. Notably, Chen et al. [10] observed that the fiber mixture exceeded most individual fibers in SCFA production, with preliminary observations suggesting that some individuals across all disease states exhibited levels surpassing expected additive contributions from individual components. We hypothesized that this mixture satisfies the mechanistic conditions underlying synergistic fermentation that we previously proposed in different fiber mixtures [7]. In that work, we argued that systematically designed fiber mixtures can provide structurally diverse configurations that accommodate the metabolic requirements of complementary bacterial groups, thereby promoting specialized niches, cooperative interactions, and reduced competitive pressures that amplify SCFA production. Given the preliminary observations from the Chen et al. [10] dataset indicating potential synergistic responses in some individuals and our interest in understanding the underlying mechanisms behind this phenomenon, this diverse dataset spanning health and disease states served as an ideal model to systematically quantify and elucidate the fundamental mechanisms driving synergistic SCFA production though designed mixtures.

The present study objective was to explore fiber synergy mechanisms by systematically quantifying synergistic interactions and exploring microbiota signatures related to enhanced SCFA production. We performed a secondary data analysis of the Chen et al. [10] dataset, comparing a four-component fiber mixture against theoretical expectations across HC and individuals with CD, UC, and PD, to identify taxonomic, metabolic, and ecological patterns associated with synergistic capacity. This work is a critical step toward understanding the mechanisms of SCFA synergy with implications for developing precision prebiotic approaches that harness cooperative microbial interactions to enhance therapeutic outcomes regardless of underlying health conditions. We hypothesized that this fiber mixture would demonstrate supra-additive synergistic SCFA production across health conditions, transcending inter-individual microbiome heterogeneity, and that specific microbial signatures would emerge as drivers of these synergistic interactions.

## 2. Materials and Methods

To quantify synergistic responses, identify associated microbial signatures, and explore cooperative microbial interactions driving SCFA synergy, we employed a secondary data analysis utilizing the previously characterized dataset from Chen et al. [10]. The original study comprised fecal specimens from 33 participants across four distinct health conditions: healthy controls (HC, *n* = 10, 58.6 ± 10.9 years), Crohn’s disease in remission (CD, *n* = 7, 40.0 ± 19.8 years), ulcerative colitis in remission (UC, *n* = 7, 50.0 ± 10.7 years), and Parkinson’s disease (PD, *n* = 10, 62.9 ± 9.9 years) [10]. The study was approved by both the Rush University Medical Center Institutional Review Board (ORA#: 07100403, approved 26 November 2007; 12020204, approved 5 March 2012; 07092603, approved 8 October 2007; L04092807, approved 11 April 2007) and the Purdue University Institutional Review Board (IRB 1509016451, approved 16 September 2015) with informed consent obtained from all donors [10]. The in vitro fecal fermentation experiments were conducted as described by Cantu-Jungles et al. [11], with minor modifications [10]. The fiber mixture used in the study was composed of equal proportions (25% each) of four soluble dietary fibers: apple pectin (AF 710, Herbstreith & Fox KG, Neuenbürg, Germany), barley β-glucan (P-BGBM, Megazyme, Ltd., Bray, Co. Wicklow, Ireland), fructooligosaccharides from sugar cane (Nutraflora, Ingredion Incorporated, Westchester, IL, USA), and sorghum arabinoxylan (custom extraction) (see Supplementary Table S1 for more details). These fibers were selected based on their diverse structural characteristics to provide a blend of different soluble fiber substrates. Fermentation experiments were conducted for 12 h using individual fibers as well as their mixture with the fecal inocula from the different donors, and SCFA analysis was performed on supernatants from fermentation samples using gas chromatography (GC-FID 7890A; Agilent Technologies, Santa Clara, CA, USA) on a fused silica capillary column (No. 40369-03A; Nukon Supelco, Bellefonte, PA, USA) to measure acetate, propionate, butyrate, and total SCFA concentrations as previously described by Chen et al. [10].

To evaluate whether the mixture yielded higher SCFA concentrations than individual fibers and quantify synergistic effects, we conducted comparative analyses by first calculating descriptive statistics (means and standard deviations) grouped by health condition and fiber type. Synergy was expressed as the relative difference between observed mixture SCFA and the SCFA production’s expectation, reported as a percentage, using the following formula:

$$Synergy\;(\%)\;=\;\frac{\left({Observed}_{mixture}-\;{Expected}_{additive}\right)}{{Expected}_{additive}}\times 100$$

The “*Expected*” value represents the average of SCFA production from the four individual fiber components of the mixture for each participant, equivalent to the sum of individual components in equal proportions (25% each):

$${Expected}_{additive}=\;\frac{{SCFA}_{Fiber\;1}+{SCFA}_{Fiber\;2}+{SCFA}_{Fiber\;3}+{SCFA}_{Fiber\;4}}{4}$$

Differences between observed and expected values were assessed, and to avoid overstating small or unstable effects, we used uniform thresholds across all measurements to filter out the data when within the measurement error (|*Observed* − *Expected*| ≤ 0.5 mM) and below the minimal detection limit of 1.0 mM. After the filtering step, no statistics were performed if the number of remaining samples per condition and per SCFA were lower than 3 (*n* < 3). 

SCFA data were analyzed using paired, two-tailed tests to account for repeated measurements from the same donors across fiber treatments. All analyses were performed in OriginPro 2023b (OriginLab Corporation, Northampton, MA, USA) [12], as follows:

(1) Comparison of fiber treatments: Within each health condition (HC, PD, CD, UC), observed SCFA values from the fiber mixture were compared with each individual fiber using Tukey’s method for multiple pairwise contrasts. For visualization purposes, the mean SCFA value of the four individual fibers was calculated for each donor.
(2) Observed versus expected mixture performance: A donor-specific expected SCFA value was calculated as the mean of the four individual fibers. Observed and expected mixture values were compared using paired tests within each condition and each SCFA type, with Benjamini–Hochberg correction applied to control false discovery.
(3) Synergy quantification: Synergy was defined as the percentage deviation of the observed mixture value from each donor’s expected value, as described before. After applying threshold-based filtering, synergy values for acetate, propionate, butyrate, and total SCFAs were compared across health conditions. Because filtering produced unequal sample sizes, *p*-values were adjusted using the Holm–Šidák method.

To identify taxonomic correlations of synergistic responses, we calculated Spearman correlations between SCFA production, alpha-diversity metrics, and the synergistic response percentages. The alpha-diversity metrics analyzed included initial baseline measurements, post-fermentation measurements, and changes between baseline and post-fermentation states (∆) for Shannon diversity, richness, and evenness indices. Additionally, differential abundance analysis comparing individuals without synergy versus those exhibiting synergy was performed using Analysis of Composition of Microbiomes with Bias Correction (ANCOM-BC; implemented via the q2-composition plugin (version 2024.10.0) in QIIME 2 (version 2024.10.1), Bioconductor, CO, USA) [13], with statistical significance set at *p* < 0.05 and appropriate corrections applied for multiple testing. To further explore microbial interactions associated with SCFA synergy, taxa–taxa correlation matrices and co-occurrence networks were generated using the SCNIC plugin in QIIME2 (SCNIC, version 2020.10, run within QIIME 2 version 2024.10.1) [14] and visualized in Cytoscape (version 3.10.3, Cytoscape Consortium, San Diego, CA, USA) [15]. Networks were based on Spearman correlations, with edges representing significant associations (positive or negative) and node size proportional to centrality, allowing qualitative comparison of connectivity patterns and identification of key hub taxa across synergy conditions.

## 3. Results

### 3.1. Comparison of Fiber Mixture to Individual Fibers

**Figure 1:**
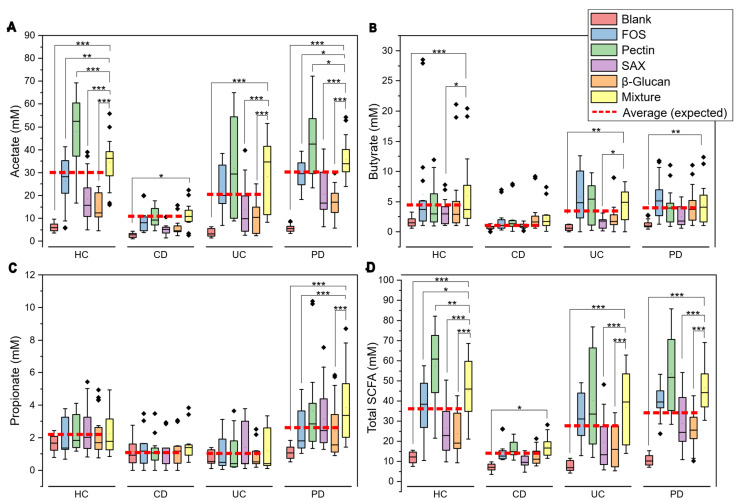
SCFA production by health condition for individual fibers and their mixture. Panels (**A**–**D**) show acetate (**A**), propionate (**B**), butyrate (**C**), and total SCFA (**D**) in millimolar (mM) across HC, PD, CD, and UC. The dashed red line indicates the expected production based on individual fiber contributions. Asterisks denote statistical significance (* *p* < 0.05, ** *p* < 0.01, *** *p* < 0.001).

Comparative analysis of SCFA production in the fiber mixture versus its individual components stratified by condition showed that the fiber mixture consistently outperformed most individual fibers across health conditions (Figure 1). The fiber mixture and pectin were the only fiber sources to show consistently higher SCFA production across all the health conditions in comparison to individual fibers and their average. While the mixture did not surpass pectin’s individual performance, this one being the most effective single fiber, no statistically significant difference existed between pectin and the mixture in total SCFA production across all conditions except in HC.

**Figure 2:**
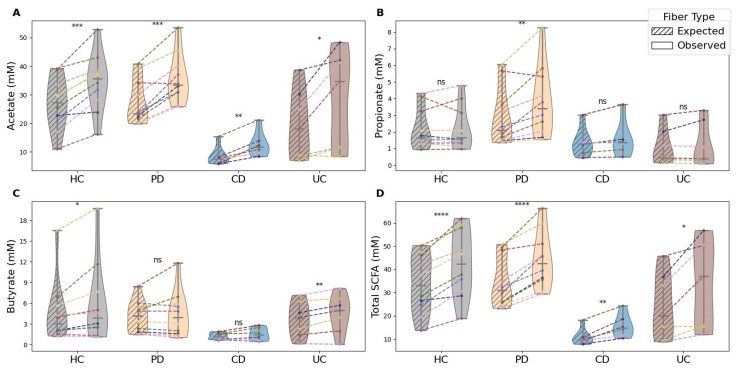
Observed versus expected SCFA production by health condition. Panels (**A**–**D**) show acetate (**A**), propionate (**B**), butyrate (**C**), and total SCFA (**D**) in mM from fecal fermentations under HC, PD, CD, and UC. Solid colors indicate observed production with the fiber mixture; hatched bars indicate expected levels from individual fiber contributions. Dashed lines connect donors to illustrate observed–expected pairs, with each line color representing a different donor. Asterisks denote statistical significance for observed versus expected comparisons (* *p* < 0.05, ** *p* < 0.01, *** *p* < 0.001, **** *p* < 0.0001; ns, not significant).

Notably, the fiber mixture elicited beyond-expected SCFA production in all four health conditions studied (Figure 2): HC, CD, UC, and PD. When examining patterns by specific SCFA types, condition-specific tendencies could be noted. For acetate, observed SCFA production for the mixture exceeded expected levels in all conditions (HC [*p* < 0.001], PD [*p* < 0.001], CD [*p* < 0.01], and UC [*p* < 0.01]). For propionate, the mixture led to unexpectedly high levels in PD (*p* < 0.01), whereas for butyrate, the pattern was significant in HC (*p* < 0.05) and UC (*p* < 0.01). Finally, for total SCFA production, beyond-expected levels were observed across all conditions: HC (*p* < 0.0001), PD (*p* < 0.0001), CD (*p* < 0.01), and UC (*p* < 0.05). Overall, regardless of health condition, the mixture effectively promoted beyond-expected levels of SCFA, hereafter referred to as synergistic effects.

### 3.2. Synergy Quantification

The median percentage synergy (%) was calculated across all health conditions, and was positive for acetate, propionate, butyrate, and total SCFAs, confirming the supra-additive responses of the fiber mixture (Figure S1, “All conditions”). The fiber mixture synergistically increased total SCFA production by 32.8 ± 20.1% on average, across donors and conditions, reaching up to 85.7 ± 14.9% (UC donor 5). Within-condition comparisons showed some SCFA-specific differences. In HC, propionate displayed the lowest synergy and was significantly lower than acetate and butyrate (*p* < 0.01). In PD, butyrate tended to be the lowest (*p* < 0.01). No within-condition differences reached significance in CD or UC. When pooling all donors (“All conditions”), butyrate was significantly lower than acetate (adjusted *p* < 0.05); no other pairwise comparisons were significant. Distribution spreads that include occasional negative values (synergy < 0) indicate inter-individual heterogeneity despite a consistent positive central tendency (Figure S1).

### 3.3. Microbial Correlates of Synergistic SCFA Production

**Figure 3:**
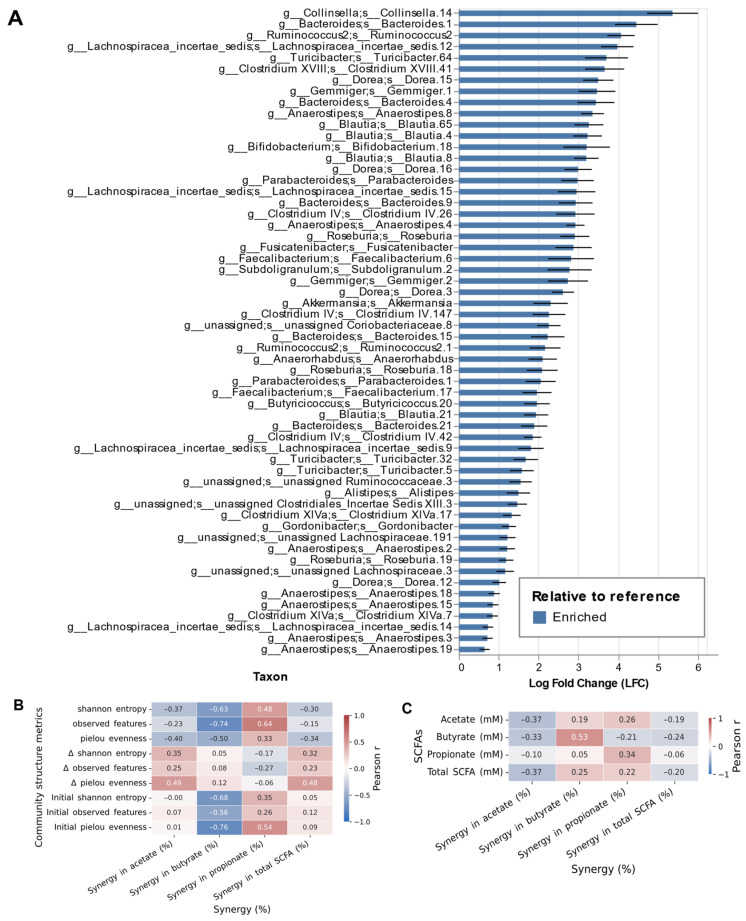
Taxonomic signatures, alpha-diversity, and SCFA production metrics associated with synergy. (**A**) ANCOM-BC differential abundance analysis of enriched species in donors with synergy versus no synergy. Bars show log fold change with standard error; positive values indicate enrichment in the synergy group. (**B**) Spearman correlations between percent synergy (acetate, propionate, butyrate, total) and alpha-diversity metrics (Shannon diversity, richness, evenness) at baseline, post-fermentation, and change from baseline (Δ, post-fermentation minus baseline). Tiles display correlation coefficients; asterisks denote significance; no statistically significant correlations were detected (*p* ≥ 0.05). (**C**) Spearman correlations between percent synergy and absolute amounts of SCFA concentrations (mM). Tiles display correlation coefficients; significance is annotated as in panel (**B**).

To identify microbial signatures that could lead to synergistic responses, we performed differential abundance analysis using ANCOM-BC. Individuals exhibiting synergistic responses showed promotion of multiple genera within the *Lachnospiraceae* and *Ruminococcaceae* families, with additional increases from *Bacteroidaceae* and *Coriobacteriaceae* (Figure 3A). Species from genera such as *Collinsella*, *Bacteroides*, *Ruminococcus*, *Turicibacter*, *Dorea*, *Gemmiger*, *Anaerostipes*, *Blautia*, *Bifidobacterium*, *Parabacteroides*, *Roseburia*, *Faecalibacterium*, and *Subdoligranulum* showed log fold changes ranging from 1 to 5.7, with *Collinsella* being the most promoted taxon. No specific species were significantly depleted in individuals with synergy compared to those with no synergy. Network analysis further revealed distinct co-occurrence patterns (Figure S2). Acetate synergy involved dense positive clusters centered on *Ruminococcaceae* and *Clostridiaceae* together with *Bacteroidaceae*; propionate synergy showed mixed associations in which *Lachnospiraceae* acted as a major hub alongside *Bacteroidaceae*, *Erysipelotrichaceae*, and *Verrucomicrobiaceae*; and butyrate synergy was dominated by highly connected *Lachnospiraceae*–*Erysipelotrichaceae* modules embedded within broader *Clostridiaceae*, *Coriobacteriaceae*, and *Fusobacteriaceae* clusters (Figure S2). These patterns support the idea that there could be enhanced cooperative interactions among key fermentative taxa as drivers of SCFA synergy. Notably, the synergistic boost in SCFA production was not correlated with any alpha-diversity metric (Figure 3B) nor with absolute SCFA amounts (Figure 3C).

## 4. Discussion

Extending our previous findings, which demonstrated that strategic fiber combinations elicit synergistic responses [7], we quantified and evaluated the synergistic interactions and potential cooperative bacterial associations in health and disease states by employing a secondary analysis from Chen et al. [10]. In vitro SCFA production profiles of a soluble dietary fiber mixture composed of equal parts of apple pectin, barley β-glucan, fructooligosaccharides, and sorghum arabinoxylan in microbiotas from individuals with CD, UC, PD, and HC were used to identify SCFAs and the community and taxonomic signatures associated with such interactions, and were compared across health conditions.

Our findings demonstrate potential for synergistic SCFA production when using fibers as a mixture across health and disease states. The mixture consistently outperformed most individual fibers, enhancing SCFA production beyond what individual components provide alone. Notably, synergistic interactions with the mixture boosted total SCFA production by an average of 32.8% (and up to 80% in some donors) beyond the expected levels. Remarkably, this synergistic potential was preserved across all health conditions studied and not related to the absolute production of SCFA. In fact, the highest synergistic responses occurred in individuals with PD (for propionate) and CD and UC (for acetate), which are conditions characterized by low SCFA [16], highlighting the therapeutic relevance of fiber mixtures in restoring gut microbiome metabolic function. These suggest that cooperative microbial interactions can be harnessed regardless of the initial microbiota context, whether healthy or disease-associated.

Previously, we showed that designed mixtures can support gut microbial diversity [7], and thus, here we have tested if alpha-diversity signatures would be associated with synergistic SCFA boosts. Interestingly, no correlations were found with any metric tested, suggesting that the mechanisms explaining synergy likely depend on specific taxon–taxon interactions rather than on alpha-diversity alone. In fact, differential abundance analysis demonstrated multiple bacterial taxa within both *Lachnospiraceae* and *Ruminococcaceae* families being more represented in participants exhibiting synergistic responses. These families include key members of *Clostridium* clusters XIVa and IV, major butyrate producers that closely associate with intestinal epithelial cells, support gut homeostasis through metabolic and immunomodulatory functions, and colonize interfold niches adjacent to the epithelium, positioning them for cooperative substrate use and cross-feeding [17]. Network analyses further reinforced that both *Lachnospiraceae* and *Ruminococcaceae*, together with other dominant families such as *Clostridiaceae*, *Bacteroidaceae*, *Fusobacteriaceae*, *Erysipelotrichaceae*, and *Verrucomicrobiaceae*, occupied central hub positions within the acetate-, butyrate-, and propionate-associated synergy networks, exhibiting strong positive co-occurrence patterns with other fermentative taxa. This high connectivity indicates *Lachnospiraceae* and *Ruminococcaceae* taxa as potential mediators of the cooperative microbial interactions that drive the synergistic enhancement of SCFA production. Markedly, Chen et al. [10] reported *Lachnospiraceae* being less abundant in HC and PD compared to UC and CD [16,18], which could justify the observed higher synergy in these conditions. Overall, the interactions of particular taxa, rather than a community diversity signature, support the synergistic interaction for SCFA production, and are conserved across health and disease states.

We have previously proposed [7] that strategically designed fiber mixtures provide diverse carbohydrate structures that better accommodate the metabolic requirements of complementary bacterial groups compared to individual fibers. Cooperative interactions may arise as a consequence of the exploitation of microbial specialization preferences and reduction in competitive pressures [19,20], ultimately leading to amplified SCFA production beyond expected levels. Our results align with these ideas by demonstrating synergistic SCFA production across health and disease states with the enrichment of potential complementary taxa. The hypothesis that bacterial taxa combinations are critical to determine SCFA production is corroborated by Fernando et al. [18] with single culture observations that demonstrate that inter-genus bacterial combinations produce significantly more SCFAs than intra-genus combinations due to the reduced metabolic competition and enhanced cooperative interactions [21]. Furthermore, our results contrast with Lancaster et al. [19], who found that fiber mixtures showed smaller or intermediate effects compared to individual fibers, suggesting that the effectiveness of synergistic outputs likely depends on the strategic composition of mixtures rather than simple substrate source diversity.

This study shows that fiber mixtures can act synergistically across different health conditions by quantifying responses at the individual level, using appropriate statistical analyses, and probing mechanisms with differential abundance and network approaches. Several factors should be considered when interpreting these findings. These represent plausible mechanisms requiring validation, not definitive characterization. In vitro fermentation excludes host physiology, mucosal interactions, and immune modulation regulating in vivo SCFA production. Moreover, differential abundance analyses remain correlative, and future in vivo studies with metabolomic validation are needed to establish causality and definitively characterize the mechanisms driving synergistic SCFA production. Finally, the synergistic interactions noted here were observed in a single mixture formulation and cannot be extrapolated to other mixtures. Looking forward, understanding the mechanistic insights into cooperative fiber interactions could enable the rational design of optimized fiber formulations with enhanced therapeutic potential, informing evidence-based approaches to develop superior prebiotic therapies tailored to a wide range of health conditions for improved clinical outcomes.

## 5. Conclusions

This study represents the first systematic quantification of fiber mixture synergy in SCFA production across health conditions, demonstrating that a four-component blend (apple pectin, barley β-glucan, fructooligosaccharides, sorghum arabinoxylan) synergistically enhances SCFA production by 32.8% above expected levels, independent of health status. Our results reflect synergy driven by cooperative microbial interactions likely mediated by *Lachnospiraceae* and *Ruminococcaceae*, along with other central hub families such as *Clostridiaceae*, *Bacteroidaceae*, *Erysipelotrichaceae*, *Fusobacteriaceae*, and *Verrucomicrobiaceae*, and occurring even in dysbiotic communities, independent of microbial diversity. These findings suggest that fiber mixtures may offer therapeutic potential for restoring gut metabolic function to beyond-expected levels regardless of the baseline health condition.

## Data Availability

The data supporting the findings of this study are openly available in the GitHub repository, https://github.com/Galeano-Garcia-GS/Synergistic-SCFA-production-by-Fiber-Mixture (accessed on 12 November 2025). The repository contains all relevant datasets, including raw and processed data, as well as scripts used for data analysis and visualization.

## Supplementary Materials

The following supporting information can be downloaded at https://www.mdpi.com/article/10.3390/nu17243904/s1, Table S1: Composition and characteristics of individual and mixed dietary fibers used in in vitro fecal fermentation experiments, Figure S1: Percent synergy in SCFA production by health condition and pooled donors; Figure S2: Taxa–taxa correlation networks representing microbial associations under synergy conditions for each SCFA.

## Abbreviations

The following abbreviations are used in this manuscript:

SCFAs	Short-chain fatty acids
HC	Healthy controls
CD	Chron’s disease
UC	Ulcerative colitis
PD	Parkinson’s disease
