# Supplementary material for: New Insights into Synergistic Boosts in SCFA Production Across Health Conditions Induced by a Fiber Mixture
Source: Nutrients. 2025 Dec 13;17(24):3904. doi: 10.3390/nu17243904 (PMC12735490; doi:10.3390/nu17243904)
Supplement: Supplementary file 1 [file nutrients-17-03904-s001.zip › nutrients-4008489-supplementary.pdf]

Fiber Component	Source Material	Product/Brand	Purity	Key Compositional Elements	Structural Features
Fructooligosaccharide (FOS)	Sugar cane	Nutraflora, Ingredion, USA (Westchester, IL)	>95% fiber	Oligosaccharide chains	Fast-fermenting fiber; commonly used prebiotic
Beta-glucan (β-glucan)	Barley	P-BGBM, Megazyme, Bray, Ireland	>95% fiber	Linear alternating β-1,3 and β-1,4 glucan linkages	Hemicellulose; known butyrate-promoting fiber; low gas production relative to SCFA
Pectin	Apple	AF 710, Herbstreith & Fox Inc., Werder, Germany	>95% fiber	Galacturonic acid, rhamnose, galactose, arabinose	Acetate producer (~80% acetate of total SCFAs); easily fermenting soluble fiber
Sorghum arabinoxylan (SAX)	Sorghum	Custom extraction [10]	>95% fiber	β-1,4 linked xylan backbone with arabinose, xylose, galactose, glucuronic acid branches; fairly simple branched pattern with monosaccharide arabinose units on highly substituted xylan backbone	Hemicellulose; low gas production; selective propionate promoter without inducing large gas amounts; better tolerability in vivo; best tolerated fiber (lowest gas:SCFA ratio)
Fiber Mixture	Combination of all four above	Combined components in equal proportions (25%)	>95% each component	Combined composition of all four fibers	

Supplementary Table S1. Composition and characteristics of individual and mixed dietary fibers used in in vitro fecal fermentation experiments. The table summarizes the four individual fermentable soluble dietary fibers (>95% purity each) and their equal-proportion mixture (25% each component) used to investigate microbiota responses across health conditions. Each fiber component is characterized by distinct compositional elements and structural features: FOS (fast-fermenting prebiotic), beta-glucan (butyrate promoter, low gas production), pectin (acetate producer, ~80% of total SCFAs), and sorghum arabinoxylan (lowest gas: SCFA ratio, high tolerability).

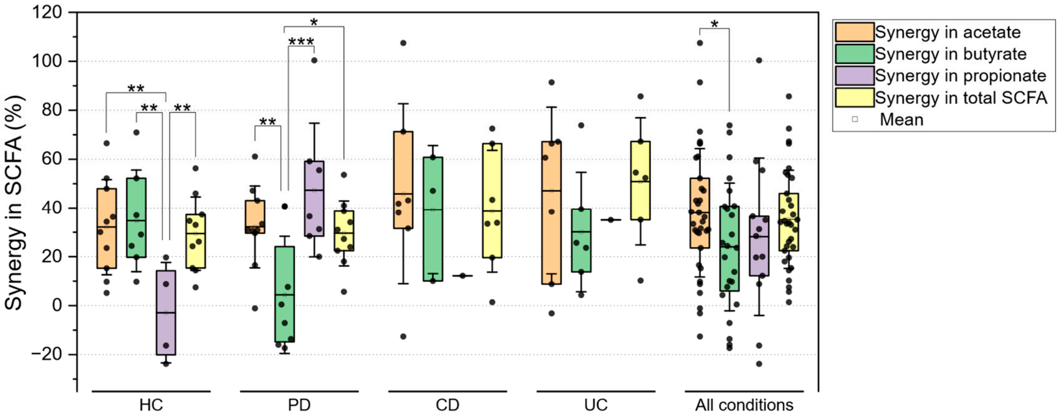

Supplementary Figure S1. Percent synergy in short-chain fatty acid (SCFA) production by health condition and pooled donors. Box plots show % synergy for acetate (orange), butyrate (green), propionate (purple), and total SCFAs (yellow) after 12-h fecal batch fermentations with the four-fiber mixture in HC, PD, CD, UC, and the pooled "All conditions" group (n per cohort in Methods). Horizontal brackets denote pairwise comparisons among acids within each cohort and in the pooled group (*$p<0.05$, **$p<0.01$, ***$p<0.001$, ****$p<0.0001$).

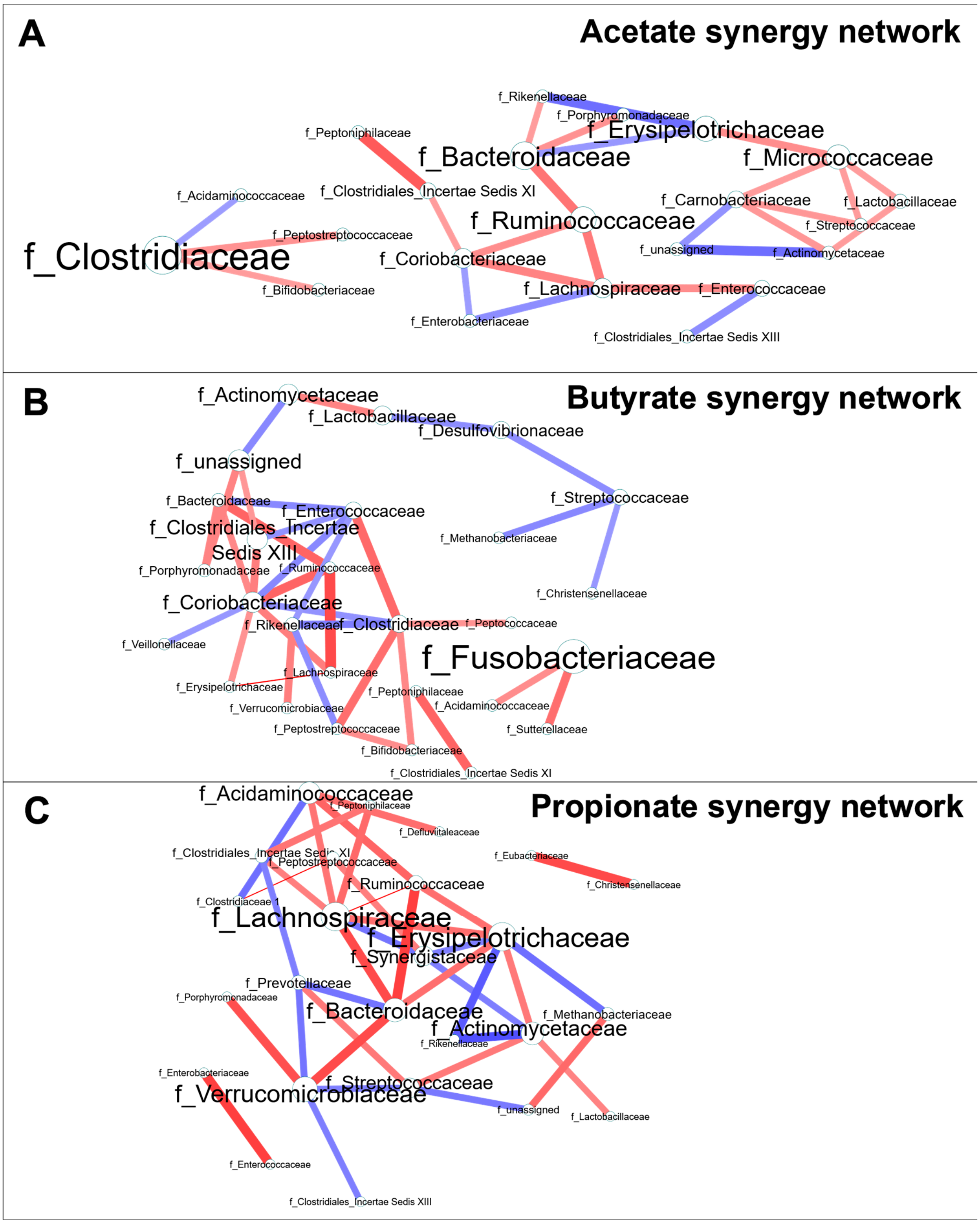

Supplementary Figure S2. Taxa–taxa correlation networks representing microbial associations under synergy conditions for each SCFA. Networks were constructed using the SCNIC plugin in QIIME 2 (SparCC correlations, $r \geq 0.40$; $n = 34$) and visualized in Cytoscape using the Compound Spring Embedder (CoSE) layout. Nodes correspond to bacterial families; edge color gradients indicate correlation coefficient (r, red = positive, blue = negative), edge width represents correlation strength ($|r|$), and node size reflects centrality (importance within the network). Each panel represents microbial association patterns in donors exhibiting synergy for a specific SCFA. (A) Acetate-synergy network, (B) Butyrate-synergy network, (C) Propionate-synergy network.
